# Observations on the Medical Topography, Climate, and Endemic Influences of South Alabama

**Published:** 1846-12

**Authors:** James C. Harris

**Affiliations:** Wetumpka, Alabama


### WESTERN JOURNAL

OF

MEDICINE AND SURGERY.

DECEMBER, 1846.

Art. I.—*Observations on the Medical Topography, Climate, and Endemic Influences of South Alabama.* By James C. Harris, M.D., of Wetumpka, Alabama.

Aware of no systematic attempt since the work of Dr. Heustis, published at Cahawba, in 1825, at a description of the causes, symptoms, and treatment of our autumnal fevers, and being thoroughly impressed with the importance and correctness of the remark of Malte Brun, "that not only the best observations upon climate often lose half of their value, for the want of an exact description of the surface of the country, but that the diseases incident to particular localities cannot be properly comprehended or treated," I have been induced to offer to the profession of the South the following facts, observations, and deductions, under a hope that should they fail to enlarge our views concerning the etiology or treatment of fever, they may serve to bring about a more correct understanding of the subjects involved, and answer hereafter as a neucleus, in the hands of some one more able to do the subject justice, in the formation of a more comprehensive treatise on the diseases of the South.

The Southern portion of Alabama, of which it is our intention more particularly to treat, lies entirely between the parallels of 30° 10′ and 32° 30′ north latitude. The extreme southern limit bordering on the gulf of Mexico, for the space of 50 or 60 miles, is low and level, and covered with pine and cypress. The remainder, off the water courses, consists of rolling uplands interspersed with prairies. The soil of the ridges and uplands is generally sandy and unproductive, but throughout a large portion, particularly along the margins of rivers and creeks and in the prairies, it is most excellent, covered in some places in a state of nature with a dense and impenetrable growth of cane, and in others, clad in a forest of oak, hickory, dogwood, magnolia, &c.

The western limit of the great Atlantic plain at Columbus, Georgia, is very distinctly marked by a ledge of primary rocks, which recede at that point to the northwest through Alabama and Mississippi, until the Atlantic plain is merged in the Mississippi valley. Among the physical features that characterize this alluvial zone, the northern limit of which in passing near Wetumpka and Tuscaloosa is but little elevated above the Gulf of Mexico, and to which it gently slopes down, are that it is composed entirely of the tertiary and cretaceous deposits, and abounds in the sands and marls and organic remains of that series of formations, being furrowed with deep ravines and sluggish streams, interspersed with extensive morasses and swamps, and having wide arms of the Gulf of Mexico extending far inland. Thus this portion of the State presents a great southern inclination of surface to the action of the sun, filled with all kinds of organic matter undergoing decomposition, and creating one among the most extensive hotbeds of malaria known within the geographical limits of the Union.

The mean annual temperature of our climate may be stated at about 64° Fah., and although not subject to so great a thermometrical range as many other countries, still the variations from heat to cold are sudden and frequent, often rendering fires during the summer and fall months after sun down and blankets at night indispensable to comfort, and that after a day when the heat has ranged from 72° to 90°. The winters are so mild that the streams are never frozen over, and the heats of summer are greatly mitigated by refreshing breezes from the Gulf.

The principal rivers are the Mobile, the Alabama, and their tributaries, the Tombigbee, the Cahawba, the Talapoosa, and the Coosa, the two latter forming the Alabama. The Mobile, about 50 miles above the city of Mobile, is formed by the union of the Tombigbee and the Alabama, which latter is also a considerable stream, and is navigable for vessels, drawing from five to six feet water, to Claibourne, 60 miles above its junction; one hundred and fifty miles further, viz: to the mouth of the Cahawba, it has four or five feet of water, and from thence to the junction of the Tallapoosa and Coosa, and up the latter to Wetumpka it is navigable for light draught steamboats with few exceptions at all seasons of the year.

Mobile, the emporium of the State, is situated on the west bank of Mobile river, at its entrance into Mobile Bay, in latitude 30° 40″ north; it is laid out upon a beautiful and extended plain, elevated some fifteen feet above the highest tides, open to refreshing breezes from the bay and commanding a fine prospect.

The other towns on the Alabama, or immediately above the limits of the Atlantic plain, of any importance are St. Stephens upon the Tombigbee, Tuscaloosa upon the Black Warrior, Claibourne, Cahawba, Selma, and Montgomery upon the Alabama, and Wetumpka upon the Coosa.

As a general rule, South Alabama is but indifferently supplied with permanent springs, some of the most fertile districts being almost entirely destitute. This is more especially true of the counties of Green, Marengo, and Sumpter, and the prairie portions of Montgomery and Lowndes. In these districts the artesian auger has been used to supply this deficiency.

From what has already been said it will be perceived that South Alabama proper consists almost entirely of Atlantic plain and slope, and that the extreme limits of the former terminate upon a circle drawn from Mobile and extending some hundred and fifty miles from the Gulf of Mexico, into the interior, embracing within its circumference St. Stephens, Cahawba, Selma, &c. This region, over which are scattered hill and valley, woodland and prairie, is emphatically that of long moss, and mosquetoes, the former gradually extending its mournful drapery, and the latter their musical companionship to the extreme boundary of the Atlantic slope.

To the mind of the believer in the malarial origin of fever, there is perhaps no particular region of the south or southwest that furnishes so many proofs in support of his opinion, as the one we have just described, and none, with whose history we are familiar, that affords so many bright examples in elucidation of some of the most obscure and long disputed points connected with this subject.

The greatest wonder with us is not that the atmosphere of the region we have been describing should during the summer and fall months become unhealthy, but that it should not actually become unfit for respiration, and instantaneously destructive of life. The question then is not whether our atmosphere becomes adulterated or sensibly altered, which all of the least observation are compelled to admit, but what kind of alteration it is that it undergoes, or what additional element it is that it receives. This as yet is a subject that has eluded the research of the most scientific. From many facts within our knowledge we are irresistibly driven to the conclusion, that for the development of yellow fever there must be some sensible alteration in the atmosphere of the locality, aside from that necessary to the production of other forms of bilious disorder. In a communication upon this subject, received by the writer from Dr. Edward Gault, of Selma, bearing date July 1st, 1846, it is remarked that during the summer of 1820 the heat was not greater than the present and many others, where the mercury ranges from 80° to 95° in the shade; with seasonable showers; nor did the yellow fever, or any other malignant epidemic prevail that summer in Mobile, or at any other point upon the Alabama river, equal to Cahawba, where the disease was of a high grade of remitting fever, in many instances terminating fatally upon the 3d, the 5th, and 7th days. It was rarely attended with black vomit, but frequently with collapse, and a chocolate, or brown cadaverous hue. Up to the fall of 1821, there had been no navigation of the river by steamboats, but since that time this means of communication has been introduced and has increased every year.

The situation of Cahawba is described by Dr. Gault as being low, upon the north bank of the Alabama river, which at high tides overflows the entire town. East, and above the town, the Cahawba river enters the Alabama, and upon the north, one mile, a creek enters the former; on the west is a considerable extent of marshy ground, giving vent to the water from a higher table of lands, two or three miles off in that direction. Across the Alabama river, at no great distance, was a pond, or lagoon, covering some 20 acres, which has since been drained.

In addition to the above causes, a large amount of alluvial soil was exposed to the action of the sun, from the cutting down of the timber and cane, and clearing up and putting into cultivation large districts of the surrounding country, and this, with the additional fact that but few, if any, of our citizens were acclimated, being generally from the old settled parts of the northern and middle States, a scarcity and an inferior quality of provisions, together with the disappointments and vexations incident to settling a new country, constitute some of the remote and predisposing causes which in our opinion, operated most powerfully in the production of the epidemic under which we labored.

In 1824 the yellow fever appeared in Selma, and that section of the country known as the Pleasant Valley, ten or twelve miles north; one case under Dr. Phillips terminated fatally on the third day after black vomit, and several cases under my inspection on the fifth and seventh days after the same, some in collapse. There was no yellow fever in Mobile at the time.

The planters of the Valley attributed the fever to the extensive rot and decay of the roots of the cane brakes, which took place about that time.

In the year 1826, the yellow fever prevailed in Mobile, and the steamboats were so infected with the disease that several towns refused their stopping. Montgomery and perhaps also Selma, would not receive their sick. I have been credibly informed by the citizens of Mobile, that during the prevalence of this fever not one lived who attempted to remain in the city after an attack; that there was only a single case of death after removal to the country; and that not a solitary case of fever occurred in the country in any family among whom the sick were removed.

As regards the cessation of fevers at the beginning of frost, it appears from my observations that frost does not always check the prevailing fever of any type, unless when accompanied by a corresponding change in the atmosphere, and that change I presume would answer without frost. I have seen the fevers of the fall type in Tennessee continue until the middle of the following February.

Since residing upon the Alabama river, I have observed no cause so certain to produce a sickly summer, as an overflow or inundation in the spring, and the "*later* the *worse*."

Dr. Heustis, in his work before alluded to, remarks that the low grounds and swamps adjacent to the Alabama and Cahawba rivers, were frequently inundated during the spring of 1821, so that it was late in May before many of the farmers upon the rivers had an opportunity of planting, owing to the highness of water. A considerable rise in the river took place in July, and such was the quantity of rain that fell early in the summer, that many of the farmers were entirely frustrated in their attempts at planting, the earth being so completely wet and inundated, that the seed rotted in the ground; so that many were obliged to plant the same field three or four times, and then in several instances were doomed to lose their labor, and abandon the undertaking as hopeless.

Such indeed was the situation of affairs in many instances upon the river; in others less injury was sustained, and upon the uplands remote from the river the crops came forward with considerable certainty; even there, however, crops of corn and cotton were injured by the excessive rains, and replanting became a business of equal necessity.

The average range of the thermometer during the month of June, was at 7 A.M., 73°; 2 P.M., 85°; and 9 P.M., 77°; and throughout the month of July the average temperature, as indicated by the same thermometer, was, at 6 A.M., 71°; at 3 P.M., 84°; and, at 9 P.M., 75°; making the mean temperature throughout the day and night 76°.20.

'In Alabama,' continues the same author, 'we certainly have a much longer continuance of hot weather, as well as a higher range of temperature during the summer months, than is found to prevail in the State of Pennsylvania. I have frequently observed the thermometer as high as 90° or 96° in the passage of a tolerably cool house. This circumstance creates among us a greater liability to summer and autumnal diseases, than the inhabitants of more northern countries.'

In addition to the foregoing causes it may be remarked that the temperature throughout the months of August and September continued about equal to that of July, towards the latter part of which month, and when the rains had measurably ceased, and the water of the ponds and marshes had considerably evaporated, and the river had fallen within its banks, the work of putrefaction went on with rapid strides, and disease began to make its appearance.

As it is a well established fact that a country clothed with a dense forest and luxuriant vegetation, from the protection furnished the surface of the earth by the thick foliage of the trees, and the evaporation from the leaves of the different vegetables, is *colder* and more *healthy* than one that is being cleared up, it follows as a matter of course that the climate of particular localities in Alabama, during the process of clearing, has undergone a considerable change in this particular, and that it is upon this hypothesis that we are enabled, to a considerable extent, to account satisfactorily for the various epidemics that have successively visited various sections of our State; nor have we the shadow of a doubt that circumstances such as we have detailed, together with the nature of the seasons, have been the remote cause of the fevers that have prevailed at different times at Cahawba, Selma, Mobile, St. Stephens, Montgomery, and Wetumpka, as well as the other towns and neighborhoods in South Alabama. That they will forever recur, at intervals, under similar circumstances, we are as thorougly satisfied and have as good reasons for believing.

After examining the rise and progress of yellow fever, throughout the southern portions of the United States, and comparing the facts thus acquired with the following table, it will appear that there is no well authenticated account of yellow fever prevailing as an epidemic further from the seacoast upon a straight line, than one hundred and fifty miles, and that this distance has only been acquired in four instances, under peculiar circumstances, as at Cahawba, Alabama, and Natchez, Mississippi.

GEORGIA.

*Savannah*—Lat. 32° 8′ north; long. 4° 10′ west. Tide water.

*Augusta*—Lat. 33° 24′ n., long. 4° 45′ west. Distance from tide water 110 miles.

*Columbus*—Lat. 32° 30′ n.; long. 8° west. Distance from tide water 165 miles.

ALABAMA.

*Mobile*—Lat. 30° 40″ n.; long. 11° 10′ west. Tide water.*

*St. Stephens*—Lat. 31° 30′ n.; long. 11° 5′ w. Distance from Mobile 64 miles.

*Cahawba*—Lat. 32° 20′ n.; long. 10° 10″ west. Distance from Mobile 130 miles.

*Wetumpka*—Lat. 32° 30′ n.; long. 9° 15′ west. Distance from Mobile 175 miles.

*Tuscaloosa*—Lat. 33° 15′ n.; long. 10° 38′ west. Distance from Mobile 180 miles.

LOUISIANA.

*New Orleans*—Lat. 29° 57″ n.; long. 14° 13′ west. Tide water.†

MISSISSIPPI.

*Natchez*—Lat. 31° 22′ n.; long. 14° 30′ west. Distance from New Orleans 105 miles.

As there are other places in the same latitude equally surrounded with all the local causes known, under favorable circumstances, to produce malaria in great abundance, it follows as a matter of course, that there must be some other action in the atmosphere, which being added to its already existing malarious constitution, renders it capable of producing the disease.

To illustrate our position more clearly, we will now endeavor to ascertain what this sensible alteration and its cause is, and then see if yellow fever does not always appear under exactly the circumstances known to be in existence at the places, and in the seasons that have been found by experience necessary to its production.

---

* From Mobile to Mobile point 30 miles.

† From New Orleans to the Gulf of Mexico 55 miles.

We believe that it is a well established fact that yellow fever, so far as the United States are concerned, has never appeared as an epidemic in a higher latitude than 40° north, and even then but seldom, and immediately upon or very near the sea shore, as at New York, Philadelphia, Baltimore, Richmond, &c.

As there have as yet been but few hygrometric experiments made in the United States, and those, so far as we are able to ascertain, confined to its northern portions, remote from the ocean, we labor under great disadvantages in the investigation of this subject, but although in a great measure divested of necessary data, we have some observations bearing immediately upon the point which we think are of so clear a nature, that they will be sufficient to convince the most skeptical of the truth of the following propositions; namely: That if yellow fever does not always originate in low southern latitudes, immediately upon the ocean, or large bodies of water, under high, long, and continued heats, it is for the want of a corresponding alteration in the dew point; that these circumstances when they do occur below the line of 33° of latitude in Georgia, 32° in Alabama, and 31° 30′ in Mississippi, and south and south west of these points, are most generally attended with a high dew point; and that it is only when acting together, that they possess the power of producing the disease under consideration in an epidemic form.

Fortunately, remarks Dr. Lee, as a general rule in our climate (that of New York) the dew point is many degrees below the temperature; it is but rare indeed that they nearly or quite coincide, and when they do such weather is called sultry or close, and its depressing influence upon the system is too well known to be described; the very color of the skin, to say nothing of the languor of the mind, and the debility of the muscular system, shows that the blood does not undergo the proper change in the lungs. We also find that highly malignant fevers prevail where the dew point is below 60°, and that in high northern latitudes, and the northern and middle portions of the United States, except at a few points below 40°, and upon the ocean, the heat is never sufficiently high for any length of time to carry the dew point up to the 60th degree. This, however, unfortunately for us is not so in our southern latitudes; here, within the tropics, and the country bordering thereon, we find as a general rule a hygrometric condition of the atmosphere vastly different from that which prevails in the temperate zones. With a high summer temperature, we have also a high dew point. Captain Alexander states that during his voyage to the river Gambia in Africa, the thermometer, on the 6th of October, stood at 80° in the shade, whilst the hygrometer stood at 70°. At another time the thermometer was 84°, and the hygrometer 79°.

The annual average mean of the hygrometer in the Island of St. Vincent, one of the Carribee Islands of the West Indies, in latitude 12° 23′, is always above 60°, and the average heat is proportionably high. Hence we infer that in Havana, Vera Cruz, and all other places where yellow fever and other high grades of bilious fever are prevalent, the mean heat and the dew point must be correspondingly high; and that for sometime before and during the prevalence of yellow fever, they approximate very closely to each other, and produce a close, sultry, and oppressive state of the atmosphere.

In the West Indies, yellow fever is endemic, and prevails from six to nine months of the year. When it becomes epidemic in the city of Havana, it is always under high and long-continued thermometrical and hygrometrical influences. These, in connexion with already existing malarious causes, never fail to produce the worst forms of the disease. It generally becomes epidemic in the city of Havana, which is in latitude 23° 12′, about the first or middle of May, and five or six weeks afterwards in the city of New Orleans. Shortly after its appearance in this latter city cases are also reported at Mobile, and afterwards at Savannah.

In its spread from New Orleans up the rivers throughout Louisiana and Mississippi, and from Mobile and Savannah, the same ratio of progression is observed, until it has reached the extreme limit of the Atlantic plain, where it ceases, from the well known fact that the epidemic constitution of the at-atmosphere necessary to its production never passes this boundary.

Indissolubly connected with the foregoing causes, are some others that must exercise a controlling influence in the production and extension of our endemics; and of these perhaps heat, elevation, and winds, exert as great an influence as any others. In Alabama as we advance from the sea shore to the interior or hilly regions, bordering upon or beyond the extreme limits of both the Atlantic plain and slope, the summer heat is increased, and as we return to the sea board upon the same plain, the cold of winter is moderated in the same ratio.

From what has been said it is evident that the dew point and the thermometrical heat upon an island under the equator, or nearly so, as at St. Vincent, will at all seasons be nearly coincident; and that when the daily or monthly mean heat should steadily range from 80° to 95° in the shade, and the hygrometer indicate the dew point as correspondingly high, yellow fever, the highest grade of malarial disease known to these islands, or in the southern portions of the Union, would be developed.

This atmospheric condition, aided perhaps by other causes heretofore enumerated, being communicated through the influence of the land and sea breezes from the Island of Cuba, where it nearly always prevails, to New Orleans, Mobile, and Savannah, when it finds sufficient local food in these cities must produce, though perhaps in a less aggravated form, the same character of disease.

It has been demonstrated by actual experiment,* that when

---

* British Army Meteorological Report.

the atmosphere is loaded with moisture, the heat of the same and the dew point more nearly approximate. Then with a temperature at New Orleans or Mobile throughout the months of June, July and August, ranging from 85° to 90°, with a corresponding elevation in the dew point, we could not expect anything else, provided moisture and vegetable matter were in sufficient quantities, but the prevalence of all the high grades of febrile disease. It must however be borne in mind that whilst the mean summer heat is greatly modified upon the coast by the sea breeze, the dew point owing to great moisture of the air has been ascertained to be frequently elevated. As we advance upon the Atlantic plain, and until we pass a certain elevation, the first is slightly increased, and the latter diminished, until we pass its confines, where they are no longer capable of producing high grades of malarial disease.

Although the relative difference between the mean summer temperature, upon the sea coast and the interior, has been shown to be greater at Tampa Bay, Florida, than at Jefferson Barracks, Missouri,* still we do not wish this to be taken as giving a fair representation of the fact upon that subject, so far as the great Atlantic plain in Georgia, Alabama, and Louisiana is concerned, as it is a well established position from innumerable thermometrical observations made at different times, and at various places, that the annual mean summer temperature is several degrees lower on the sea coast, upon the bays, the interior lakes, and for some distance up the large water courses than at other points upon the same plain, and remote from these. In fact only a small part of the southern regions of South Carolina, Alabama, Louisiana, or Mississippi, below the 33d, 32d, and 31st degrees of latitude, appear to be sufficiently elevated to cause a diminution in the summer heat; but on the contrary, off the large water courses the reverse obtains. The thermometer

* Forry on Climate.

ranges in the interior from 98° to 72°; whereas on the sea board it scarcely ever exceeds 91° or 92°. This difference was observed between Charleston and Columbia in 1808; it has also been noticed between Savannah and Augusta, Wetumpka and Mobile, and Natchez and New Orleans; and is greater in proportion to elevation and removal from the sea shore, and beyond the influence of the sea breeze, and other sources of moisture. In assuming the above position we do not wish to be understood as meaning to advance the idea, that the annual temperature is increased from the equator to the poles; but merely as stating a physical fact, in relation to the summer temperature of the regions in which yellow fever most generally, if not universally, delights to dwell.

From all that we can learn or have observed, we feel perfectly satisfied that the result of the combined operation of the remote causes heretofore enumerated, are such as to create a predisposition to disease, which only requires some exciting cause to develope the various grades of our autumnal fevers; and that they are all, from the simple intermittent to the more formidable yellow fever, the result of physical causes alone acting upon constitutional predisposition.

And here for the present we conclude. The treatment of fever remains to be considered, and that shall be made the subject of a future communication.

Wetumpka, July, 1846.

